# Public education during epidemics of infectious diseases: A national mixed-method study with parallel convergent design in a low and middle-income country

**DOI:** 10.1371/journal.pone.0328451

**Published:** 2025-08-01

**Authors:** Saber Azami-Aghdash, Salar Mohammaddokht, Maryam kashani, Nima Pourgholam, Mohsen Nouri, Elaheh Nasiri

**Affiliations:** 1 Tabriz Health Services Management Research Center, Tabriz University of Medical Sciences, Tabriz, Iran; 2 Student Research Committee, Tabriz University of Medical Sciences, Tabriz, Iran; 3 Nursing and Midwifery Care Research Center, School of Nursing and Midwifery, Iran University of Medical Science, Tehran, Iran; 4 School of Nursing and Midwifery, Iran University of Medical Science, Tehran, Iran; 5 Spiritual Health Research Center, Iran University of Medical Sciences, Tehran, Iran; 6 Department of Health in Disasters and Emergencies, School of Health Management and Information Sciences, Iran University of Medical Sciences, Tehran, Iran; 7 Medical Philosophy and History Research Center, Tabriz University of Medical Sciences, Tabriz, Iran; Lorestan University of Medical Sciences, IRAN, ISLAMIC REPUBLIC OF

## Abstract

**Introduction:**

Proper and effective public education during epidemics of infectious diseases can have a key effect in controlling epidemics and reducing their complications. Therefore, the present study aims to assess public education methods during infectious disease epidemics from the perspectives of both the public and experts in Iran.

**Method:**

The present study is a mixed-methods (quantitative-qualitative) with a parallel convergent design conducted in 2024 in Iran. The public’s views on the effectiveness, strengths, and weaknesses of each method used for educating people during epidemics, with a focus on the COVID-19 epidemic, were collected through a self-development valid and reliable questionnaire (with closed and open-ended questions). Quantitative data was analyzed using SPSS:16 software. In the qualitative section, data were collected by semi-structured interviews and manually analyzed using content analysis methods.

**Results:**

Television (79.9%), social networks (78.8%), and websites (78.5%) were introduced as sources that have performed best, provided various information and education, had a high impact on the audience, and successfully gained people’s trust during epidemics of infectious diseases. In the qualitative section, the majority of participants identified in-person training as the most effective method of educating people. Utilizing the capacities of mass media and providing accurate information to the public were strengths, while dissemination of false and unscientific information and lack of trust in relevant institutions were identified as weaknesses in educating people during epidemics of infectious diseases.

**Conclusion:**

In this study, an attempt was made to provide comprehensive and sufficient information for decision-making and effective planning for public education in the next epidemics of infectious diseases.

## Introduction

Throughout history, humans have seen many epidemics of infectious diseases, the spread of these epidemics has caused fear in humans and affected their physical and mental health [1–5]. According to the definition of the World Health Organization (WHO) and the Center for Disease Control and Prevention (CDC), an epidemic means the occurrence of a disease or complication in a certain population more than expected [6,7]. An epidemic is the rapid spread of an infectious disease to most of people in a specific population in a short period, usually two weeks or fewer [7,8]. Epidemic diseases can cause the death of many people and create large economic and social problems [9–11]. Despite the change in the pattern of diseases from infectious diseases to chronic diseases [12], in recent decades, the world has witnessed several epidemics of infectious diseases, including SARS, MERS, COVID-19, and N1H1 influenza [13]. The 2003 SARS epidemic caused more than 8,000 cases in 29 countries, including 29 in the United States. There were 774 deaths from SARS (although no cases of SARS have been reported anywhere in the world since 2004) [14]. Since 2012, a total of 2,613 cases of Middle East Respiratory Syndrome (MERS) have been reported worldwide, with 943 associated deaths, for a case-fatality ratio (CFR) of 36%. The majority of these cases have been reported from Saudi Arabia, with 2,204 cases and 862 associated deaths (CFR: 39%) [15]. The latest pandemic was the COVID-19 disease, which was first detected in China and in less than three months, it spread all over the world and led to the creation of a global pandemic [16–18]. According to WHO, until January 2024, the total number of coronavirus patients in the world is more than 750 million people, of which more than 7 million people have died due to this disease [19]. Among the people affected by the COVID-19 disease, the European region (more than 230 million people), the Western Pacific (more than 200 million people) and the American region (more than 190 million people) have the most people and the African region with about 10 million people, have the lowest rate of COVID-19 disease [20]. Also, among the deaths caused by this disease, America and Europe are at the top (each with more than 2 million deaths), and Africa has the lowest number of death due to the COVID-19 pandemic with less than 200,000 deaths [21].

Since the effects of epidemics can be seen in all aspects of people’s lives [9,22], during the epidemic crisis of infectious diseases, different programs and interventions are designed and implemented in various fields to control and prevent it [23]. Different countries adopted multiple policies in this regard, for example, in Australia, traditional methods of influencing citizens’ behavior such as laws, fines, and taxes were used [24]. The results of Anderson et al.’s study (2020) show that the government’s communication strategies are vital to informing the public about the best methods to prevent infection [25]. Educating public is also one of the most important and priority fields because through education, people’s awareness and health literacy have increased, and they can better cooperate in disease prevention and control [26]. Training personal hygiene, social distancing and staying at home, and using personal protective equipment (PPE) are the most common training during epidemics of respiratory infectious diseases [27,28]. Labaf and colleagues (2021) discussed educational and communication methods during the COVID-19 pandemic in Iran, highlighting the sharing of experiences through virtual platforms, providing daily statistics, and educating the public through credible resources, including e-learning for the community. They emphasized that part of the healthcare system should take on the responsibility of providing education and establishing communication [29]. Also, the study of Mohammaddokht and colleagues (2024) points to the effect of providing training from the media and mass communication tools during the Covid-19 epidemic [30]. In Azami and colleagues’ study (2022), the role of non-governmental organizations in disease prevention and control through education, fundraising and attracting international cooperation is also mentioned [31]. Wing-Keung (2020) suggested that strengthening public education, apart from strengthening basic infection control practices such as wearing masks and hand hygiene, regular conversations should be through classrooms or through mass media should be organized to convey to the people the concept of cough etiquette, social distancing, home quarantine, avoiding gatherings and stopping eating wild animals appropriately and according to the circumstances [32,33].

Due to the spread of epidemics of infectious diseases in recent years and their many complications, officials, and stakeholders in the field of health and health education used different methods to train people in order to reduce the effects of epidemics. However, the review of available texts and evidence shows that there are no sufficient studies in the field of evaluating the methods of training public during the crisis of epidemics and the effectiveness of this training from the point of view of people and experts. Therefore, according to the importance of the training and its costs, the effectiveness of the adopted training methods and the views of the people and experts on them can be useful and provide key information for the policy. It should be presented and provided to decision makers and decision makers, especially for the design, implementation and management of intervention programs for possible future crises and epidemics. Also, the evaluation of educational methods can identify the challenges and shortcomings of implemented programs in order to prevent the recurrence of these cases in the future and to take steps to improve educational methods. Therefore, the present study was conducted to evaluate public education methods during epidemics of infectious diseases from the perspective of people and experts in 2023 in Iran.

## Materials and methods

Study Design: This study is a mixed-method (Quantitative-Qualitative) of the parallel convergent model type of alignment design categories. In these studies, the goal is to obtain different but complementary data on a subject [34].

### Methodology of the Quantitative Section of the Study

#### Type of Study, Setting, and Participants.

The first section of the present study is a cross-sectional study conducted in 2023 in Iran. The target population for this part of the study was people aged 15 and above in all cities of Iran. The inclusion criteria for this section included being at least 15 years old, having basic literacy skills, and having experienced at least one type of health education during the epidemic of infectious diseases. Additionally, individuals who were unable or unwilling to participate in the study were excluded.

#### Sample Size and Sampling Method.

To determine the sample size, Morgan’s table was used, and based on the statistical population, a sample size of 396 individuals was calculated [35]. To enhance the study’s power and reduce the impact of sample dropout, 20 percent was added to the sample size, resulting in a final sample size of 476 individuals for the study. For sampling, a convenience sampling method was employed. The study instrument, a questionnaire, was distributed to primary health care facilities throughout various Iranian cities, enabling health workers to administer it to clients of all ages, genders, and occupations.

#### Data Collection Tool.

The tool used in this section of the study was a questionnaire developed by the research team (Appendix 1). To prepare the initial questions for the questionnaire, seven semi-structured interviews were conducted with experts in the field of the study (medical education, health education, epidemiology, healthcare management, and health education specialists from the Health Deputy Office) to identify and list the initial questions. After conducting the interviews, the research team members immediately transcribed and analyzed the texts and statements of the participants, and based on the results of the analyses, the initial questions for the questionnaire were extracted. To qualitatively assess the face validity of the tool and determine the time required to complete it, feedback was gathered from the target group (face-to-face interviews with 10 individuals from the study’s target group) to identify difficulties in understanding phrases and words, the appropriateness and relevance of the items, the potential for ambiguity, and misinterpretations of phrases or inadequacies in the meanings of words. In addition to the qualitative measurement of face validity, the quantitative method of the impact method was also used. Therefore, the questionnaire was completed by 12 people, and based on the answers of the target group on a 5-point Likert scale for each of the questions of the tool, face validity was calculated quantitatively by the researcher. Determining the content validity of the instrument, which was done to ensure that the content of the test is representative of the construct it is claimed to measure, was also determined by two quantitative and qualitative methods. In the qualitative review of the content, various experts related to the subject were requested to submit their corrective views in writing. In this study, two indexes, content validity ratio (CVR) and content validity index (CVI) were used for quantitative analysis of content validity. To determine the reliability of the final version, the internal consistency of the questionnaire was done using the semi-structural method and Cronbach’s alpha (α = 0.87). Finally, the questionnaire used in the study had 14 questions, of which 4 were about demographic information and 10 were about study objectives. All the questions of this questionnaire were finally approved by the experts with CVI = 0.93 and CVR = 0.91.

#### Data collection process.

The questionnaire used to collect information was designed in Persian and in both paper and electronic form and was provided to the target group in different urban and rural areas by the research team and also with the help of experts from primary health facilities. For available people, the printed version of the questionnaire was used. In such a way, one person from the research team provided the paper-based questionnaire to the participants and after coordination, he delivered the completed questionnaire. Collecting data from other people, especially people who were present in other cities or villages, was done anonymously and based on the internet and online platforms by sending emails, social networks and links to people. This link was created through the website (https://survey.porsline.ir/). People who did not respond were periodically followed up. The first follow-up was done four weeks after sending the first email/link and the second follow-up was done eight weeks after sending the first email/link. To facilitate data collection, we tried to get help from people (students and colleagues) living in different cities.

#### Data analysis method.

For data analysis, SPSS Statistics version 16 was used. In the descriptive section, frequency (percentage) was used for qualitative variables, and for quantitative variables, mean (standard deviation) was used if the distribution was normal, and median (interquartile range) was used if the distribution was not normal, to summarize the information. According to the data normality test results, parametric statistical tests of T-test and Pearson’s correlation coefficient test were used. A p-value less than 0.05 was considered significant.

### Methodology of the qualitative part of the study

#### The type of study, setting and its participants.

The second part of this study is a qualitative study with a descriptive qualitative approach. The descriptive qualitative approach is one that focuses on examining the characteristics of a phenomenon rather than explaining the causes or underlying mechanisms. This includes the collection and analysis of data in the form of words, images, or other non-numeric forms of information [36]. In the present study, the perspectives of experts, the public, and officials regarding methods for educating people on effectiveness, strengths and weaknesses, and strategies for enhancing the effectiveness of each of the methods used for public education during epidemics of infectious diseases were examined separately. The criteria for the entry of officials and experts into this section of the current study included: having a responsibility related to educating the public during epidemics, possessing sufficient knowledge and information in the field of public education during epidemics (such as publishing articles or books in this field), and having the ability and willingness to participate in the study. The criteria for the entry of individuals into the current study included being at least 15 years old, having at least basic literacy skills, and having experience receiving at least one type of health education during the outbreak of infectious diseases.

#### Sample size and sampling method.

The sampling method of the study to select seven health care professionals and eight public health education experts was based on purposive sampling. In the selection of the participants, an incongruous method (with maximum diversity) was used based on the type of expertise, field of study, level of education, work experience and place of service. Available sampling method was also used to select people.

#### Data collection methodology.

Data was collected through semi-structured interviews with experts and knowledgeable individuals in the field of public education during epidemics. At this stage, a semi-structured interview guide was used to conduct the interviews. This guide was developed using the insights of experts in the subject, literature reviews, and input from those experienced in qualitative studies. The necessary data for the current study was gathered through in-person interviews with experts from 2 May 2023–15 November 2023. Initially, information about the interview topic was provided to potential participants via phone or email, and necessary arrangements regarding the location and time of the interviews were made. To better manage the interview sessions, an interview guide designed by two members of the research team was utilized (Appendix 2). This guide included six open-ended questions, and before finalizing it, two pilot interviews were conducted to identify potential issues. After the pilot interviews, the interview guide was finalized with minor changes. The language of the interviews and focus group discussions (FGD) was Turkish and Persian. Initially, the questionnaire was translated into English and then from English to Persian to verify the accuracy of the translation. Finally, with slight modifications, it was translated back into English. Each session was managed by two members of the research team; one member facilitated the sessions while the other served as a note-taker, recording key points from the participants’ comments. Additionally, if clarification was needed based on the notes taken, feedback was provided to the participants immediately after the discussions ended. All sessions were recorded with the participants’ permission using a recording device, and the recordings were transcribed and analyzed immediately after each session. The qualitative data collection process (interviews with experts) took approximately 7 months. The total duration of the interviews was 302 minutes, with the longest interview lasting 84 minutes and the shortest lasting 10 minutes (an average of 20.13 minutes). The interviews continued until data saturation was reached. In this study, the researchers achieved relative saturation after conducting 12 interviews, but to ensure adequacy, interviews continued up to the 15th interview. Additionally, to obtain supplementary information, interviews with two experts were repeated. The information from all interviews was utilized for analysis.

In order to enhance the richness of the collected data and utilize triangulation in data collection, a FGD was held with the participation of five officials from the education sector during epidemics. To better manage the group discussion, a semi-structured guide designed by the research team members was used. The session took place at the Education and Health Promotion department of Health Deputy Office of East Azerbaijan Province. The language of the FGD was Turkish, but the implementation was conducted through Turkish to Persian translation. The FGD was facilitated by three members of the research team, with one member leading the session while the other two served as note-takers, recording key points extracted from the participants’ comments. Additionally, if clarification was needed based on the notes taken, feedback was provided to the participants immediately after the session concluded. The FGD lasted for 1 hour and 24 minutes. With the participants’ permission, the session was recorded using a tape recorder and was transcribed and coded immediately after the meeting.

In another section of the present study, public opinions were collected using three open-ended questions designed in the questionnaire (quantitative section of the study). This questionnaire was made available to the public in both paper and electronic formats. The paper-based questionnaire was provided in person to individuals who were physically accessible, while a link to the electronic questionnaire, designed on the Porsline platform, was shared with those who were geographically inaccessible. Participants in this section of the study wrote their responses to the open-ended questions either in the physical questionnaire or typed them in the electronic version. In this section, 197 individuals who provided sufficient and relevant responses to the open-ended questions in the questionnaire were selected (15 responses were excluded from the study due to irrelevance and inappropriateness).

#### Data analysis methodology.

For the analysis of all qualitative data across the three sections of the study, content analysis was employed, which is a method for identifying, analyzing, and reporting the themes present within the text [37]. The coding of the data was conducted independently by two researchers, and any discrepancies were resolved through discussions between the two researchers or by consulting another member. The stages of data analysis and coding were as follows: initially, immersion in the text and multiple readings were conducted, and the initial codes derived from the text were extracted. Then, based on these codes, themes were developed, and the codes were classified within these themes. To ensure the reliability of the codes, a second coder was utilized. All stages related to coding were performed manually.

#### Rigor.

To enhance the robustness and accuracy of the study results, four criteria proposed by Guba and Lincoln were utilized. Acceptability and confirmability: For this aspect, long-term engagement and review by colleagues and participants, as well as the input of experts and knowledgeable individuals, were employed. After the sessions concluded and the participants’ opinions were summarized, a summary of the participants’ statements was presented to them based on notes taken during the sessions to correct and clarify any errors or ambiguities. Dependability: For this aspect, two individuals were used for coding to assess the reliability of the information. Transferability: For this aspect, the opinions of experts and knowledgeable individuals were utilized, along with purposive sampling, to evaluate the applicability of this information in different times and places.

### Ethical considerations

The study was approved by the Research Vice-Chancellor of Tabriz University of Medical Sciences Ethics committee with the code of ethics IR.TBZMED.REC.1401.822. Written informed consent was obtained from all the participants in this study. The principles of confidentiality regarding the anonymity of the participants in the study and maintaining the confidentiality of information were observed.

## Results

### Quantitative results

In the current study, a total of 278 individuals participated, yielding a response rate of 0.6. The mean age of the participants was 33.9 years, with females comprising the majority of the sample (61.9%). Most participants held a bachelor’s degree (43.2%), and the majority of them were students (23.6%) (Table 1).

**Table 1:** General characteristics of the participants in the quantitative part of the study.

Variable		Frequency (%)
**Gender**	Female	172 (61.9).
	Male	106 (38.1).
**Education level**	Bachelor	120 (43.2).
	Diploma	53 (19.1).
	Master’s degree	43 (15.4).
	Post graduate	28 (10.1).
	Under diploma	18 (6.4).
	Ph.D.	16 (5.8).
**Job title**	Student	65 (23.6).
	Employee	61 (22.1).
	Housekeeper	50 (18.1).
	Other	48 (17.4).
	Health system employee	42 (15.2).
	Teacher	10 (3.6).

**Fig 1:**
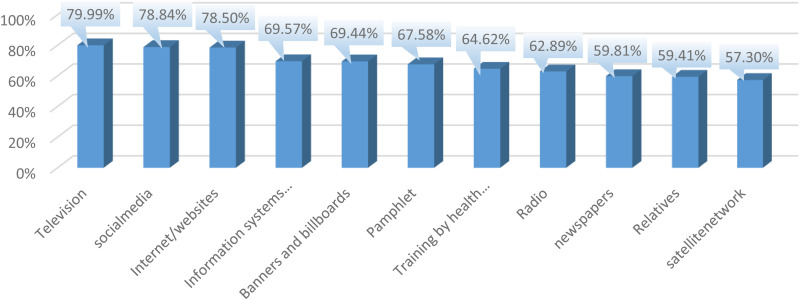
The number of overall scores of information sources for training people in epidemics.

Results of the evaluation of scores from various information sources across the four fields of ‘performance, impact on the audience, gaining public trust, and the richest source’ showed that, overall, television, social networks, and the internet received the highest scores from the public, with 79.99%, 78.84%, and 78.50%, respectively (Fig 1).

**Fig 2:**
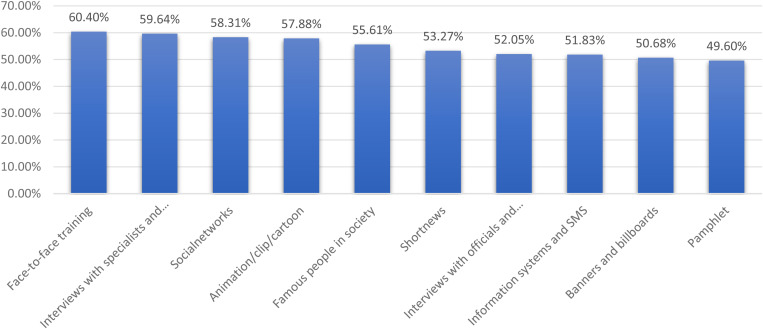
The effectiveness of teaching methods to people in epidemics.

Additionally, in the field of the most effective methods of educating the public, in-person training (60.40%), interviews with health specialists and experts (59.64%), and content creation and dissemination on social networks (58.31%) accounted for the highest percentages (Fig 2).

Statistically, there was a significant relationship between gender and the amount of using satellite network and relatives to receive information and training (p-value = 0.001). No significant difference was observed in other information sources. Also, the correlation results between age and the average score of information sources showed that among different information sources, age is related to the Internet (r = −0.196, P = 0.001), social networks (r = −0.218, P < 0.001) and newspapers (r = −0.128, P = 0.035) has an inverse significant relationship.

### Qualitative results

In the qualitative section of the present study, interviews were conducted with 15 experts and officials in the field of public education, with the majority of interviewees being male (60%). More than 70% of the interviewees held doctoral degrees. Additionally, the majority of participants (67.7%) were faculty members at universities.

#### Most common educational resources and methods.

Based on the data collected from interviews and FGD, 145 themes were identified in the section on resources and methods for educating the public during the epidemic of communicable diseases. According to the opinion of the participants in the present study, social media and educational and health experts were considered the most common sources of information used by the public during the epidemic of communicable diseases.

Additionally, according to most participants in the present study, social networks were identified as one of the most common sources of information used by the public during the COVID-19 pandemic. In this regard, one of the participants stated that:

*“...Currently, the main source that people focus on and use is social media...” [Faculty Member/ 6*^*th*^ *Interview]*

In this context, another interviewee referred that

*“...The level of trust that people have in these social networks is, for various reasons, higher than that in mass media and even health facilities...” [Faculty Member/ 2*^*nd*^ *Interview]*

Furthermore, another participant in this context noted: *“...In my opinion, the majority relied on social networks like Telegram and Instagram, and there were even a number of scientific channels created for information that provided good content...” [Health Education Expert/ FGD/ Participant NO. 3].*

Based on the data collected from interviews and FGD, 97 themes were identified in the section on training methods for the public during the epidemic of communicable diseases. According to the opinion of the participants in the present study, audiovisual educational methods and in-person community education were identified as the most effective informational methods used by the public during the epidemic of communicable diseases (Table 2).

**Table 2:** The Most Effective Methods Used by People During the COVID-19 Pandemic from the Perspective of Authorities and Experts.

Theme	Sub-theme		Frequency in Interviews and FGD
**Audio-Visual Methods**	Television*	Information subtitles on TV	4
		Local networks	1
		Short messages	1
		News	1
		Healthcare Professionals on Television	7
		Special Events Program throughout year	1
	Interview with experts		4
	Lecture		1
	Follow-up Calls		4
	Social media	Telegram Channels	12
		Celebrities’ social media pages	
	Movies, clips and video reports		7
	Animation and motion graphics		13
	Scientific documentary and infographic		3
**Written Communication Methods**	Ministry of Health Print Publications		2
	Short messages		4
	Use of banners and billboards in the city		3
	Use of educational pamphlets or brochures		3
**In-Person Training Methods**	Classrooms		1
	Training through doctors		6
	Training by a health specialist/expert		7

*Frequency of repetition in interviews and FGD

Some of the interviewees in the present study indicated that animations were among the most commonly used methods for training public during the COVID-19 pandemic. In this regard, one of the interviewees stated that:

*“...In my opinion, educational content presented as animations is better [than other methods]” [Faculty Member/ 7*^*th*^ *Interview]*

Additionally, another participant expressed

*“...Animation is a very effective method that can convey content well...” [Faculty Member/ 8*^*th*^ *Interview]*

Some participants also mentioned in-person educational methods provided by doctors and health experts as one of the most commonly used approaches for conveying education to the public during the COVID-19 pandemic. In this regard, one of the interviewees stated:

*“...One of the [types of] education is in-person education in sparsely populated areas (if the epidemic occurs in a village, one can visit house-to-house to provide necessary education and information)” [Health Education Expert/ FGD/ Participant NO. 2]*

Additionally, another participant in this context mentioned*: “...Elderly individuals prefer to receive information from a doctor or someone who provides services to them...” [Faculty Member/ 3rd Interview]*

Based on the data collected from interviews and FGD, 16 codes were identified in the section on proposed authorities for educating the public during the epidemic of communicable diseases. According to the participants in the present study, the Ministry of Health is considered the primary authority for health education during the epidemic of communicable diseases (Table 3).

**Table 3:** Proposed Trustees and Their Responsibilities during Epidemic Outbreaks from the Perspective of Officials, Experts, and the Public.

Proposed Trustees	Proposed Tasks for the Trustees of Public Education			
**Theme**	**Sub-theme**		**Frequency in Interviews and FGD**
Ministry of Health, Broadcasting, Ministry of Interior, National Crisis Management Organization, others*	Organization	Organizing specialized teams for public education		2
		Proposals for Forming a Professional Team to Prepare Training for the Public		2
		Establishing an Effective Public Relations Division		4
		Developing Advanced Information Sharing Frameworks	Designing and Implementing an Integrated Information System and Website	2
			Setting up the 4030 system	3
		Providing conditions for the implementation of interventions		2
		Identifying and employing training specialists		3
	Planning and policy making	Comprehensive and immediate planning for critical situations		2
		Determining Effective Instructional Methods	Improving people’s education methods	3
			Using efficient methods to transfer information	2
		Strategic Policymaking at the National Level	Forward-Looking Strategies for Crisis Preparedness	3
	Management and leadership	Strategic Partnership and Stakeholder Engagement for Educational Advancement		3
		Advocacy to provide training		2
		Facilitating Access to Educational Resources		3
		Delegation of planning authority to the local level		3
	Communication	Collecting and publishing reliable information		4
		Introduce of reliable sources for information		2
		Establishing a groundwork for Knowledge Sharing and Education	Setting up an Information Portal	2
			Setting up the 4030 system	3
		Identifying the state of society	Educational needs assessment	3
			Identifying the target groups of trainings	3
		External communications	Preventing the parallel work of different organizations	2
			Providing information to other organizations	2
	Monitoring and evaluation	Monitoring the accuracy of the information provided		7
		Monitoring of implemented programs		3
	Empowerment	Health workers		2
		Improving community health literacy		5
	Building trust in people			2

*Frequency of repetition in interviews and FGD

In this context, one of the interviewees stated

*“...Not only for epidemics and the Corona disease, but for every area, the main authority should be found in the Ministry (of Health and Treatment), specifically in the Department of Health Education...” [Faculty Member/ 1st Interview]*

Additionally, in the section on proposed responsibilities for the authorities educating the public, 86 codes were identified. The most important themes extracted in this domain included communication, organization, and policymaking (Table 3).

According to the results of the present study, some interviewees suggested that identifying the community’s status should be considered the most important responsibility of the authority in the field of communication during the epidemic of communicable diseases. In this regard, one of the interviewees remarked:

*“...The primary duty of the authority is to identify the situation. They should assess the views of the target community and the public...” [Faculty Member/ 6th Interview]*

In the field of strengths in educating the public during the epidemic of communicable diseases, a total of 260 codes were identified, which after analysis were reported in the form of 7 main themes and 51 sub-themes (Table 4).

**Table 4:** The Most Important Strengths of Education Expressed by Officials, Experts, and the Public During the COVID-19 Pandemic.

Theme	Sub-theme		Frequency in Interviews and FGD
**Educational resources**	Utilizing of varied instructional resources		6
	Utilizing social media capabilities		21
	Utilizing the Ministry of Education’s capabilities		3
	Utilizing the capabilities of environmental advertising		4
	Training by health staff	Behvarz*	15
		Moragheb-e-salamat*	6
	Utilizing the capacity of mass media	Television	23
		Radio	5
	Existence of useful websites	World Health Organization website	2
**Educational methods**	Implementing phone-supported health literacy initiatives		5
	Provision of a dedicated phone number (4030) for information and support		2
	Implementing differentiated instruction for varied learner groups		6
	Utilizing printed educational materials such as banners, posters, and pamphlets for instructional purposes		6
	TV educational programs	Interview with health experts	5
		Movies, short videos and animation	4
		News	6
	Providing face-to-face training		9
	Sending educational SMS from the Ministry of Health		2
**People’s culture**			2
**Educational content**	Providing the accurate information to public		22
	Wide access to training		8
	Variety of trainings		8
	Training development aligned with target group needs		6
	Public health education in plain language during the corona crisis		2
	Effective notification and warnings		6
	Continuous provision of training		9
**Information dissemination strategies**	Rapid and consistent information dissemination		7
	Utilizing audio-visual media		
	Setting up the 4030 system		
	Utilizing social media		
	Utilizing peer group education		
**Prioritizing the disease**			3
**Management and policy making**	Human resource management	Partnering with educational professionals	8
		Benefiting from volunteer contributions	2
	Utilizing specialized frameworks	Establishment of educational content creation teams	9
		Utilizing government entity potential	2
		Organization of the educational committee at the beginning of the pandemic	2
		Establishment of NGOs	5
	Monitoring	System-wide surveillance	3
	Trust in educational authorities and health institutions		9
	Public opinion management		3
	Inter-sectoral collaboration for health		3

*A person from the Ministry of Health who works in rural and urban health facilities

The use of mass media resources for educational purposes and providing accurate information to the public in the field of educational content was among the most frequently mentioned strengths by participants in the study. In this regard, one of the interviewees stated:

*“...Considering the nature of the COVID-19 disease, we advised everyone to ‘stay at home,’ and during this critical time of the disease, our best resources were the media, whether radio or television (in the form of subtitles)” [Faculty Member/ 3rd Interview]*

Additionally, another participant mentioned

*“...Being h††onest with the public and providing accurate information about the disease and its ramifications was one of the educational strengths we witnessed during COVID-19...” [Participant number 160/ School director]*

In the field of weaknesses in educating the public during the epidemic of infectious diseases, a total of 375 codes were identified which, after analysis, were reported in the form of 7 main themes and 67 sub-themes (Table 5).

**Table 5:** The Most Important Weaknesses of Education Expressed by Officials, Experts, and the Public During the COVID-19 Pandemic.

Theme	Sub-theme		Frequency in Interviews and FGD
**Educational resources**	Insufficient virtual education in social media		4
	Multiple educational resources		5
	Not utilizing health education experts effectively	Social media	14
		National broadcasting	
	Poor performance of media such as radio, television, and social media		6
**Educational methods**	Failure to use educational methods to an appropriate extent (less use of educational videos and posters)		8
	Not utilizing face-to-face training		9
	Lack of innovation in educational methods		5
**Educational content**	Dissemination of false and unscientific information*	Rumors in social media	5
		False content provided by non-experts	21
	The Broadcasting programs	Not utilizing health education experts	3
		Weakness of radio and television in improving public health literacy	4
		Providing false information	3
	Failure to pay attention to all target groups such as the elderly and children		20
	Poor public training		4
	Generating stress in public		10
	Generating stress in public		22
	Failing to refresh training content and information		6
	Late notification to public		6
**Culturalization**	People’s wrong culture about education (people’s lack of cooperation)		12
**Institutional dishonesty and lack of openness**			6
**Management and policy making**	Erosion of public trust in institutions*	People’s lack of trust in radio and television	3
		Lack of trust in training	9
		The lack of trust of a part of the people in the government	6
	Planning	Lack of formal structure and educational programs	6
		Lack of plan for the post-crisis phase	3
		Lack of accurate implementation of existing programs	3
		Poor communication of educational programs to people	12
		Lack of preparation before the crisis	10
	Cooperation and coordination	Lack of coordination between the government and the private sector and the people	5
	Organization	Lack of a reliable source of information	7
		Absence of a single trustee to provide education and information to the community	7
		Lack of a centralized structure to educate people in the time of Covid-19	5
		Weakness of macro level policy making	6
	Human resource management	Providing false statistics and information by officials	12
		Low knowledge and information of officials	6
		Outdated expertise in health education among professionals	3
		Not utilizing design and advertising experts	5
	Financial resources	Financial burden of incorrect training	6
		Limited financial resources	
		Delayed remuneration for healthcare employees	
	Monitoring and control	Lack of monitoring of published content	8
		Lack of information monitoring system provided to people	4
**Infrastructure**	Insufficient digital infrastructure for education like internet		4
	Poor educational facilities like posters		4

*Frequency of repetition in interviews and FGD

One of the most common weaknesses identified by study participants was the spreading of inaccurate and unscientific information in the educational content domain, as well as the lack of trust in relevant institutions in health education to the public in the management and policymaking.

In this regard, one participant stated: *“In COVID-19, we did not have uniformity in educating the public. Everyone presented their information in various ways, through traditional and religious medicine etc., they mixed correct and incorrect information and delivered it to the public (the information was neither correct nor incorrect)” [Academic faculty member/ 2nd interview]*

Another participant mentioned: *“Sufficient information was not provided to the public, and everyone suggested a treatment and prevention method, and no one definitively endorsed another opinion. Also, there were many rumors about the disease, which even television contributed to spreading...” [Participant NO. 131/ Research assistant]*

In the field of presenting educational solutions to increase the effectiveness of educating the public during the epidemic of infectious diseases, a total of 336 codes were identified which, after analysis, were reported in the form of 10 main themes and 86 sub-themes (Table 6).

**Table 6:** Proposed solutions to increase the effectiveness of public education during the epidemic of infectious diseases from the perspective of officials and the public.

Theme	Sub-theme		Frequency in Interviews and FGD
**Educational resources**	Leveraging social media and online resources, such as YouTube, Telegram, and credible websites, to amplify public education		6
	Leveraging mass media presence		4
	Engaging prominent health specialists for instructional purposes		6
**Educational methods**	Diversity in educational methods	Utilizing the platform of existing social networks	20
	Use of updated and valid educational methods		
	Utilizing animation to provide information and education to people		
	Producing video content such as presenting reliable news and making educational videos		8
	Distribution of educational materials such as brochures, posters and pamphlets		10
	Providing training in other organizations such as universities and schools		3
	Strengthening the sending of educational SMS by the Ministry of Health		3
	Strengthening the provision of virtual training		3
	Strengthening the provision of face-to-face training in health facilities		13
	Utilizing the training method according to the target group		11
**Educational content**	Paying attention to the visual appeal of education		5
	Timely information to the public		3
	Use of valid and up-to-date evidence		13
	Providing training in simple language		7
	Providing attractive content based on specific target groups		13
	Education based on people’s needs		3
**Management and policy making**	Control and monitoring	Controlling educational content provided to people	8
		Control of information sources and fake news	5
	Financing	Allocation of funds for education to the community	11
		Allocation of facilities and more time for training	
		Paying health workers based on improving the health literacy of the covered population	
		Financing programs	
	Human resource management	Utilizing experts in the field of health education and their training	9
		Employing well-known and trusted people to transfer trainings	4
	Planning and foresight	Providing training before the occurrence of epidemiological crises	5
		Accurate planning and proper execution	4
	Structural changes	Creating a structured network for employing experts	10
		Creating a structure to provide education to people	
		Giving importance to the health education and promotion unit	
	Communication	Utilizing multi-layer communication and cascade training	6
		Utilizing the platform of social networks	
		Determining the educational interface in different organizations and the possibility of receiving feedback from learners	
	trust building	Building public trust	45
		Education from accurate sources and a reliable platform	
		Honesty of managers and officials in publishing correct and accurate information	
		Gaining people’s trust by providing correct information	
		Introduction of trusted sources	
	policy making	Policymaking at the macro level	3
		Utilizing past experiences	3
		Crisis management and forward-looking planning	21
	Appointment of trustee	Appointing a trustee for the management of financial resources	
		Formation of educational content production committees with the presence of relevant specialists and experts	11
		Determining the educational trustee	
		Appointing a special trustee to monitor training	
		Forming a special committee for disease control	
**Empowerment**	Increasing people’s empowerment	Reducing stress and self-control and compliance with health matters	6
	Increasing public awareness among people	Informing people about financial and economic losses	9
	Increase training with incentives	Free provision of some necessary services to encourage the community	3
	Provide pre-crisis training		5
	Improving the capacity and level of health literacy of the society		5
**Cultivation**	Promoting the culture of informing each other in the general population		11
	Promotion of education culture		
**Honesty**	Honesty of managers and officials in publishing correct and accurate information		5
	Honesty and uniform response of related people and institutions		
**social distancing**	Observing social distancing and not gathering in various ceremonies		5
**continuity**	Repeat training		5

Building trust in the management and policy-making domain and diversifying educational methods in the field of teaching methods to the public were among the most commonly mentioned strategies to enhance the effectiveness of education to the public during the epidemic of infectious diseases by the study participants.

In this regard, one participant expressed: *“People need to trust. There is currently a gap between the government/system and the people, and people do not trust the government... Firstly, trust must be established, and this is done through honesty...” [Faculty member/ 5th interview]*

Additionally, another interviewee stated: *“...Diversity is also important... We have to repeat the content to make it stick, but repeating it in the same way reduces effectiveness. We must use different methods each time to convey information...” [Health education expert/FGD/participant number one]*

## Discussion

Based on the study results, in the quantitative section, television, social networks, and websites were introduced as sources that have performed best, provided various information and education, had a high impact on the audience, and successfully gained people’s trust during epidemics of infectious diseases. In the qualitative section of the study, the majority of participants identified in-person training as the most effective method of educating people. Additionally, according to study participants, social media and educational and health experts were the most common sources of information used by people during epidemics of infectious diseases. The Ministry of Health was suggested as the primary authority responsible for training people during infectious diseases. Furthermore, communication was proposed as the most important duty for those responsible for educating the public. According to participants, utilizing the capacities of mass media and providing accurate information to the public were strengths, while dissemination of false and unscientific information and lack of trust in relevant institutions were identified as weaknesses in educating people during epidemics of infectious diseases. Building trust and diversifying educational methods were also suggested solutions for increasing the effectiveness of training people during epidemic of infectious diseases.

Television and social networks are influential and reliable sources of information for public during epidemics. Television and social networks, due to their ease of access, high transmission capacity, and numerous audience, are among the resources that have been widely used during epidemics [38]. In a study by Kim et al. (2020) that aimed to investigate the impact of news in large cities on rural areas in the United States during the COVID-19 pandemic, it was mentioned that television viewership increased during the epidemic, and the news presented on it had an impact on people’s behavior [39]. In a study by Úñez-López and colleagues (2020) that aimed to investigate the impact of the pandemic on public television in Europe, it was emphasized that television was highly effective in transmitting education and it was stated that in the future, we will see television programs that use different methods to produce educational and entertaining content and create new ways to communicate with audiences [40]. Additionally, in a study by Cuello-Garcia and colleagues (2020) that aimed to investigate the impact of social media on COVID-19 pandemic management, it was stated that social media was widely used as a source of information about the pandemic, and even doctors and researchers used social media to share recommendations and decisions made during the COVID-19 pandemic [41].

Social networks have been continuously growing and increasing in number of users since their creation, and simultaneously with the widespread use of smartphones. According to the latest statistics, social networks have more than 4.8 billion users worldwide, which is about 60% of the world’s population and more than 92% of internet users [42]. On the other hand, during the COVID-19 pandemic, from April 2022 to April 2023, more than 150 million new users joined various social media platforms. Normally, during epidemics, people use social media more than usual, which may be due to the implementation of quarantine laws and closures caused by the pandemic for entertainment or to stay informed about the latest news and receive related education [43,44]. However, social networks can act like a double-edged sword due to the vast amount of content and lack of supervision and weakness in content verification. A study by Lelisho and colleagues (2023) discusses the negative impacts such as fear, anxiety, and horror caused by news and information published on social networks on mental health [45]. Therefore, it is suggested that, considering the ease of access and widespread use of social media platforms, people’s health literacy should be improved so that they can distinguish between correct and incorrect information and better manage their health risks.

In-person training has been the most effective method of public educating during epidemics. In a study by Bernacki and colleagues (2021) that aimed to investigate the impact of COVID-19 on patients’ and healthcare professionals’ attitudes, beliefs, and behaviors towards the healthcare system and the dynamics of healthcare, it was stated that healthcare professionals emphasized the importance of in-person training and visits, and due to the challenges of remote care, they considered in-person training to be more effective than other methods [46]. A study by Vogt and colleagues (2023) also stated that in-person and in-person training has a greater impact on changing people’s behavior [47]. Providing training in person and in-person is more effective and practical than other educational methods due to the ease of understanding and expression, the possibility of questioning and answering, and preventing the deviation of education from its main path. To this end, the first step can be to provide the necessary infrastructure for in-person training. However, during the pandemic, in-person training may have many limitations. Therefore, it is suggested to provide training on observing safety protocols during in-person training and providing PPE at the training site.

Social media has been one of the most common sources of information used by people during epidemics. Social media has a wider application due to its ease of access for individuals. During the COVID-19 pandemic, social media became a vital channel for the general public to obtain information about the disease. The results of a study by Giri and colleagues (2021) highlighted the responsibility of social media and showed that there is a significant relationship between the news published on these media and people’s emotions, and that by publishing positive news about the disease, people’s fear and anxiety can be reduced [6]. Additionally, the results of a study by Liu (2022) showed that the information provided on social media affects people’s perception of the existing danger and changes their insight and behavior towards the disease [48]. However, we must consider that the use of social media may be associated with some undesirable consequences. In a study by Suarez-Lledo and colleagues (2022), it was stated that some users in the virtual space tend to criticize the measures taken to control the outbreak and question the accuracy of the information shared on social media [49]. Social media has changed our lifestyle and communication, and offers opportunities to improve many aspects of our lives, including health promotion and disease prevention. However, social media also has negative aspects. Therefore, government organizations, especially in the health-related sectors, can create timely policies to guide public opinion and behavior during epidemics and pandemics by monitoring social media.

Healthcare professionals were also one of the most common sources of information used by people during epidemics. Healthcare professionals are trusted by the public due to their high level of knowledge and skills in treating diseases. Healthcare professionals also used online networks to share information, improve awareness of health issues, and discuss challenges and practical responses to the epidemic [50,51]. Additionally, a study by Chen and colleagues (2020) stated that many healthcare professionals quickly shared their ideas, experiences, and treatment challenges in the form of online articles and blogs [52]. The epidemic has created a situation that highlights the need for rapid expansion of all healthcare professionals’ capacity to provide medical and educational care. The COVID-19 epidemic has also provided an opportunity for healthcare professionals to be more active in providing educational and medical services. It is essential that all healthcare professionals are supported and empowered to be prepared for what may come.

Based on the results of the present study, the majority of participants considered the Ministry of Health, Treatment, and Medical Education as the main authority for public educating during infectious diseases. The Ministry of Health, due to its executive and policy-making roles and as a higher authority, can be the authority for public educating during epidemics. It is suggested that this institution, using its authority, in addition to publishing up-to-date, accurate, and precise information, monitor the content published in all educational resources, including television and social networks, and take necessary actions to increase the capacity of healthcare professionals as one of the most trusted educational sources from the people’s perspective. The Ministry of Health should also play its intermediary role correctly in relation to other organizations and institutions. The meaning of intermediary and brokerage role is that the Ministry of Health should intervene in a way that brings other organizations, institutions, and ministries to work together and participate in planning and policy-making. Also, in the field of policy-making for public education, it should benefit from experts in the field of public education.

Most participants considered communication as the most important task for the authority responsible for educating people. Establishing organizational communication and the ability to acquire and disseminate up-to-date and efficient information can play an effective role in managing crises and epidemics. In a study by Vaezi and colleagues (2021), it is stated that inter-organizational cooperation is very important in crisis situations. In South Korea, cooperation between the private and public sectors in preventive measures played a significant role in the success of controlling the corona crisis, but in Iran, this relationship based on trust between the two sectors was not strong enough [53]. In a study by Zhang and colleagues (2020) that aimed to investigate how risk communication was managed during the outbreak in Wuhan, it was stated that the Wuhan government did not incorporate scientific risk communication into policy-making, and local government reports were suspended, and information propaganda was managed in an ambiguous way, which weakened public understanding related to COVID-19 and led to the spread of rumors and public panic [54]. Also, in a study by Wang and colleagues (2021), it was stated that there was significant inadequate, inconsistent, and contradictory communication about the epidemic and its risks, which was particularly prominent in the early stages of the outbreak [55]. Therefore, considering the importance of organizational communication and the information provided during epidemics, it is suggested that the educational and health authority during the epidemic provide reliable communication and information platforms to advance health goals and provide accurate information about the crisis and epidemic, and have the necessary preparedness and performance. Thus, the role of the authority in establishing effective communication between different parts of the health system seems very important. Therefore, it is suggested that the authority has sufficient power and authority to provide educational protocols and make all health institutions and organizations accountable.

The use of mass media and providing accurate information to the public have been mentioned as the strengths of public education during epidemics. The use of mass media, especially social networks, has expanded greatly in recent years. In a study by Ford and colleagues (2022), referring to the use of social media by healthcare professionals during the COVID-19 epidemic, it was stated that these individuals made extensive use of social media and tried to convey necessary training, such as the use of personal protective equipment, to the general public [56]. With the outbreak of infectious diseases, especially when traditional media do not provide relevant and up-to-date information, low-credibility information is provided by public health officials, and remote education of family health ambassadors is not enough, people use social media as an effective and accessible information source to communicate and obtain information [57–60]. In a study by Pourpir and colleagues (2023), it was also shown that students used search engines, foreign social networks, and news media to obtain information about treatment methods, COVID-19 news, and preventive behaviors, and Telegram and Instagram had the most usage [61]. Additionally, in a study by Nikbakhsh (2023), the role of online news networks and social media during the epidemic was mentioned, stating that these media have a wide range of information and provide both correct and incorrect information to the audience simultaneously [62]. Therefore, considering the widespread use of mass media, the ability to provide necessary information from these platforms, and their participation in preventive measures against diseases, it is suggested to use these media more efficiently and publish more scientific and up-to-date information to gain people’s trust, manage epidemics better, and make health goals more accessible.

The majority of participants mentioned the publication of false and unscientific information and the lack of trust of the people in the relevant institutions as the weaknesses of public education during epidemics. Due to the unknown nature of emerging epidemics and the lack of sufficient information about them, a lot of information is published from various sources, which confuses the society. In the meantime, the lack of trust of the general public in the relevant institutions regarding health and health education makes the situation more difficult and leads to the existence of rumors and false information among the people. In various studies, it is mentioned that in different countries, people are faced with a multitude of incorrect information along with limited access to reliable information sources and a lack of proper guidance [63–66]. In a study by Liu and colleagues (2024), it is also mentioned that the infodemic, with an excessive amount of information, complicated the task of distinguishing real information from false information, false information flooded social media, intensified public panic, destabilized society, and hindered people’s ability to make informed decisions about the epidemic, and significantly hindered the implementation of effective strategies to combat the spread of COVID-19 [67]. Additionally, in a study by Vaezi and colleagues. (2021), the publication of false information and the government’s performance in controlling the disease are mentioned, stating that during the COVID-19 pandemic, due to quarantine and social distancing, people used mass media more than ever, and it is necessary for the information provided in these media to have sufficient accuracy and credibility to gain public trust [53]. Therefore, considering the importance of information and education about treatment and prevention methods for epidemics and the need to create trust in the government, government organizations should adopt specific strategies to combat rumors, encourage health education experts to play an active role in promoting health knowledge, verify the accuracy of information, and guide people towards improving media literacy. These measures can strengthen cooperation between the government and the media and effectively combat false information.

According to the results of the present study, building trust and diversifying educational methods have been mentioned as strategies to increase the effectiveness of public education during epidemics. Given the unknown nature of emerging epidemics, the existence of social trust can make it easier to manage the situation and play a significant role in determining the success of public health interventions. In a study by Devine and colleagues (2024), referring to the importance of social trust and trust in the government, it was stated that political trust was a key resource for fighting COVID-19 [68]. Additionally, a study by Ji and colleagues (2024) showed that the damages caused by the epidemic in societies where citizens have more trust in their governments were significantly lower, and higher political trust helped citizens to follow reduction measures more and led to a more decisive government response [69]. Gaining people’s trust is a broad issue at various political-social levels and is achieved over time, so it is suggested that macro-policy-making institutions provide sufficient and accurate information to the people honestly. If people’s trust in the education and information provided by governments is high, the level of adherence to preventive measures will also be high [70]. The strength of the present study is that, based on the results of the primary literature review and researchers’ experiences, the present study has collected, analyzed, interpreted, and reported comprehensive and practical information from the entire country for the first time at this level and in this way. However, this study had limitations. One significant limitation was the restricted research budgets and difficulties accessing target groups in certain provinces. As a result, researchers relied on online and electronic methods to gather information. This created challenges in expanding the sample size and reaching illiterate individuals and those without smartphones, particularly in border provinces.

## Conclusion

The present study provided comprehensive and effective information from the point of view of people and health and public education experts in the field of effectiveness and application of various methods and resources of public education to people during the epidemic of infectious diseases. Based on these results, national television and social media as well as face-to-face training methods are recommended in compliance with health protocols. Also, building trust among people and controlling rumors and false information are necessary measures. The use of the results of this study by health system officials and policy makers and other responsible organizations can lead to the improvement and effectiveness of planning and preparations for future epidemics.
